# Gut microbiota composition in COVID-19 hospitalized patients with mild or severe symptoms

**DOI:** 10.3389/fmicb.2022.1049215

**Published:** 2022-12-06

**Authors:** Antonio Mazzarelli, Maria Letizia Giancola, Andrea Fontana, Pierluca Piselli, Elena Binda, Nadia Trivieri, Gandino Mencarelli, Luisa Marchioni, Antonella Vulcano, Chiara De Giuli, Concetta Panebianco, Annacandida Villani, Massimiliano Copetti, Francesco Perri, Carla Fontana, Emanuele Nicastri, Valerio Pazienza

**Affiliations:** ^1^National Institute for Infectious Diseases, INMI “Lazzaro Spallanzani”, IRCCS, Rome, Italy; ^2^Biostatistic Unit, Fondazione-IRCCS “Casa Sollievo della Sofferenza” Hospital, San Giovanni Rotondo, FG, Italy; ^3^Cancer Stem Cells Unit, Institute for Stem Cell Biologyl, Regenerative Medicine and Innovative Therapeutics (ISBReMIT), Fondazione-IRCCS “Casa Sollievo della Sofferenza” Hospital, Opera di San Pio da Pietrelcina, San Giovanni Rotondo, FG, Italy; ^4^Division of Gastroenterology, Fondazione-IRCCS “Casa Sollievo della Sofferenza” Hospital, San Giovanni Rotondo, FG, Italy

**Keywords:** microbiota (16S rRNA), COVID-19, biomarkers, SARS-CoV-2, intensive and critical care

## Abstract

**Background and aim:**

COVID-19, the infectious disease caused by SARS-CoV-2 virus that has been causing a severe pandemic worldwide for more than 2 years, is characterized by a high heterogeneity of clinical presentations and evolution and, particularly, by a varying severity of respiratory involvement. This study aimed to analyze the diversity and taxonomic composition of the gut microbiota at hospital admission, in order to evaluate its association with COVID-19 outcome. In particular, the association between gut microbiota and a combination of several clinical covariates was analyzed in order to characterize the bacterial signature associate to mild or severe symptoms during the SARS-CoV-2 infection.

**Materials and methods:**

V3–V4 hypervariable region of 16S rRNA gene sequencing of 97 rectal swabs from a retrospective cohort of COVID-19 hospitalized patients was employed to study the gut microbiota composition. Patients were divided in two groups according to their outcome considering the respiratory supports they needed during hospital stay: (i) group “mild,” including 47 patients with a good prognosis and (ii) group “severe,” including 50 patients who experienced a more severe disease due to severe respiratory distress that required non-invasive or invasive ventilation. Identification of the clusters of bacterial population between patients with mild or severe outcome was assessed by PEnalized LOgistic Regression Analysis (PELORA).

**Results:**

Although no changes for Chao1 and Shannon index were observed between the two groups a significant greater proportion of Campylobacterota and Actinobacteriota at phylum level was found in patients affected by SARS-CoV-2 infection who developed a more severe disease characterized by respiratory distress requiring invasive or non-invasive ventilation. Clusters have been identified with a useful early potential prognostic marker of the disease evolution.

**Discussion:**

Microorganisms residing within the gut of the patients at hospital admission, were able to significantly discriminate the clinical evolution of COVID-19 patients, in particular who will develop mild or severe respiratory involvement. Our data show that patients affected by SARS-CoV-2 with mild or severe symptoms display different gut microbiota profiles which can be exploited as potential prognostic biomarkers paving also the way to new integrative therapeutic approaches.

## Introduction

COVID-19 is an infectious disease caused by the Severe Acute Respiratory Syndrome Coronavirus-2 (SARS-CoV-2). It is mainly a respiratory infection characterized by a high heterogeneity in clinical evolution, with very different clinical presentations, ranging from asymptomatic or paucisymptomatic forms in most cases, to severe forms with fatal respiratory compromise (Ochani et al., 2021).

Up to date, COVID-19 severity have been associated with several risk or prognostic factors, such as comorbidities, age, sex, genetic factors, and geographical location but, predicting whether an individual will require hospitalization or respiratory support, or can recover at home, still represents a hard challenge (Yao et al., 2022). Clearly, there are likely several other factors that determine how COVID-19 evolves in the individual and they may play a role in determining disease manifestations, such as the need for respiratory support. Among these factors gut microbiota could be a valid factor to consider (Dhar and Mohanty, 2020).

Although several studies have demonstrated that respiratory infections caused by viral or bacterial pathogens are linked with variations of the gut microbiota composition (Lv et al., 2021; Mizutani et al., 2022), the role of the intestinal microbiota in the evolution of the COVID-19 is still poorly known (Zuo et al., 2020).

Pulmonary health is also affected by the gut microbiota through cross-talk between the gut microbiota and the lungs, which is designated as the “gut-lung axis” (Dang and Marsland, 2019). The gut-lung axis is supposed to be bidirectional, since microbial associated molecular patterns (MAMPs) and endotoxins can influence the lung health through circulation. Once inflammation is established within the lungs, in turns it affects the gut microbiota (Zhang et al., 2020; Qu et al., 2022). Regarding the role of gut microbiota composition on COVID-19, most of the studies studies have focused their attention on the differences between COVID-19 and non-COVID-19 patients in order to investigate the possible role of the gut microbiota in susceptibility to SARS-CoV-2 infection (Marcialis and Bardanzellu, 2019; Yamamoto et al., 2021).

In addition, other published studies have unveiled the relationship between the gut microbiota and the severity of the disease in hospitalized patients underlining how the microbiota alteration could be associated with the clinical evolution (Yeoh et al., 2021). This relationship is based on the ability of the microbiota to modulate the immune response, either through modification of the gut-lung axis (Din et al., 2021), or altering the expression of angiotensin-converting enzyme 2 (ACE2) receptors which allow SARS-CoV-2 to enter host cells (Koester et al., 2021).

To date, few studies evaluated the gut microbiota as a prognostic factor for disease progression and severity in hospitalized patients (Yeoh et al., 2021). The potential role of gut microbiota composition at the hospital admission to be proposed as one of the predictors of clinical evolution in response to respiratory viruses such as SARS-CoV-2 needs to be explored. This study aimed to investigate whether gut microbiota in rectal swabs from hospitalized COVID-19 patients along with other clinical variables, including age, comorbidities and obesity, was able to discriminate the clinical outcome during the SARS-CoV-2 early infection. In particular, we investigated the gut microbiota profiles of patients affected by SARS-CoV2 with mild or severe symptoms. In the next future the analysis of the intestinal microbiota could lead to useful potential markers of severe prognosis in order to predict if the patient will require intensive care and ventilatory support.

## Materials and methods

### Ethics statement

Informed consent from participants was not required as per the REGULATION (EU) 2016/679 OF THE EUROPEAN PARLIAMENT 138 AND OF THE COUNCIL of 27 April 2016 (concerning the protection of natural persons with regard to the processing of personal data and on the free movement of such data), and repealing Directive 95/46/EC 140 (General Data Protection Regulation), on account of the public health emergency during an infectious disease outbreak. Approval by the National Institute for Infectious Diseases Spallanzani’s Institutional Review Board was not required for the same reasons. All interventions carried out on patients were based on their needs according to clinical judgment, and were not performed for the purposes of this study. Data were analyzed anonymously.

### Study design

From May 2020 to January 2021, 600 rectal swabs were collected from patients with COVID-19 at the admission at National Institute for Infectious Diseases “Lazzaro Spallanzani” in Rome. The diagnosis has been confirmed by a SARS-CoV-2 RT-PCR positive on nasopharingeal swabs. Patients who had SARS-CoV-2 positivity with Cycle Threshold (Ct value) between 7 to 35 cycles were included into the study. Among the 600 patients hospitalized during the study period, 97 patients, admitted to the ordinary ward of Infectious Disease, were enrolled and classified in two groups according to their outcome considering the respiratory supports they needed during hospital stay: i) group “mild,” including 47 patients with a good prognosis and who never required non-invasive or invasive ventilation and ii) group “severe,” including 50 patients who experienced a more severe disease due to severe respiratory distress that required non-invasive or invasive ventilation.

No subjects had been vaccinated against COVID-19. Rectal swabs were collected at hospital admission and processed within 4 h and then stored at −80°C until analysis. All samples were collected during standard of care rounds using all the necessary precautions. Clinical records and Laboratory Information Systems (LIS) were used to retrieve patient data, including laboratory test results and clinical manifestations. 16 s rRNA sequencing was performed at Division of Gastroenterology, Fondazione-IRCCS “Casa Sollievo della Sofferenza” Hospital in San Giovanni Rotondo (Foggia).

### DNA extraction

Microbial DNA was extracted starting from 500 μl of sample using Nimbus automatic extractor (Seegene Technologies, Korea) according to the manufacturer’s protocol after treatment with 500 μl of Lysis Buffer ATL (QIAGEN, Hilden, Germany) at 56°C for 10 min with 20 μl Proteinase K (Darmstadt, Germany). Protocol used for the extraction/purification was the STARMag 96 × 4 Universal Cartige kit (Seegene Technologies, Korea). The kit utilized is applied to automatic nucleic acid purification system with the convenient handling of magnetic beads and it is intended to be used for DNA isolation from different matrix (for example serum, swabs, stool, biopsy). The purification procedure comprises four steps: sample lysis, nucleic acid bind to magnetic beads, wash debris and purified nucleic acid elution. The kit provides reagents for the purification of up 20 ug of pure nucleic acid from sample with an A260/280 ratio > 1.6–1.9 and typical concentration of 20–50 ug/ul. The elution volume was 60 ng/ul.

### Sequencing and bioinformatic analysis

The obtained DNA was quantified using a Qubit dsDNA HS Assay Kit (ThermoFisher Scientific, Massachusetts, United States) with a Qubit 4 Fluorometer. Microbiota amplicon sequencing was performed following the protocol recommended by Illumina. Briefly, amplification of the V3 and V4 region from 16S rRNA gene were obtained by PCRs using the suggested Illumina 16S Metagenomics Sequencing Workflow.^1^ The obtained fragments were sequenced with the MiSeq instrument (Illumina) with V3 reagents (600 cycle, 2 × 300 bp). Fastq raw sequencing data (deposited in ArrayExpress under the accession code E-MTAB-12236) were imported into qiime2 v.2021.2 (Bolyen et al., 2019; Estaki et al., 2020) using default parameters and then Illumina primers (forward: TCGTCGGCAGCGTCAGATGTGTATAAGAGAC AGCCTACGGGNGGCWGCAG, reverse: GTCTCGTGGGC TCGGAGATGTGTATAAGAGACAGGACTACHVGGGTATC TAATCC) were removed using q2-cutadapt plugin in trim-paired mode (Martin, 2011). Q2-dada2 plugin was utilized for trimming, denoising and filtering (Callahan et al., 2016) while q2-feature-classifier plugin (Bokulich et al., 2018) was employed for the assignment of taxonomy to amplicon sequence variants (ASVs) against the pre-trained Naïve Bayes classifier SILVA 138 99% operational taxonomic units (OTUs) full-length sequence dataset (Quast et al., 2013; Bokulich et al., 2021). Alpha rarefaction was produced with q2-diversity plugin in alpha-rarefaction, minimal sequence c ount between samples as max depth. q2-fragment-insertion plugin was used in sepp mode to produce the phylogenetic tree, against sepp-refs-silva-128, necessary in q2-diversity core-metrics-phylogenetic (executed with sampling-depth: minimal sequences count between samples). The Bray-curtis matrix from core-metrics analysis was tested with q2-diversity beta-group-significance, PERMANOVA test, *p*-values<0.05 as significant. Predictions of the functional profile of a microbial community based on 16S rRNA sequence data in R environment (Wemheuer et al., 2020) was obtained with Tax4Fun2 Pairwise comparisons of differentially abundant Kyoto Encyclopedia of Genes and Genomes (KEGG) pathways among different groups were performed using Mann–Whitney test, p-values <0.05 were considered significative, and visualized with STAMP v2.1.3 (Parks et al., 2014).

### Statistical analysis

Demographic and clinical-pathological features of COVID-19 patients with good (group “mild”) or severe (group “severe”) outcome were reported as mean ± standard deviation (SD), median along with interquartile range (i.e., first-third quartiles) and observed frequencies (and percentages) for continuous and categorical variables, respectively. The PEnalized LOgistic Regression Analysis (PELORA) was performed in order to identify clusters of bacterial populations, such that the linear combination of their abundances is differential between patients with good or severe outcome (see details in Supplementary materials and methods). All statistical analyses and plots were performed by the computing environment R (R Development Core Team 2008, version 4.2, packages: ComplexHeatmaps, supclust, ggplot2, gridExtra).

## Results

### Study population

Demographic and clinical-pathological data of the patients enrolled in this study are presented in Table 1. We enrolled 97 patients with a SARS-CoV-2 qRT-PCR positive nasopharingeal swab from a retrospective cohort of 600 rectal swabs from COVID-19 hospitalized patients. In order to evaluate the correlation between gut microbiota composition at hospital admission and clinical outcome, we divided our study population into two groups: group “mild,” with a good evolution (47/97), who never needed non-invasive or invasive ventilation, and group “severe,” who experimented a severe evolution of the disease with respiratory distress (50/97) and required non-invasive or invasive ventilation. As showed in Table 1, in group “severe” pneumonia and hypertension rate was statistically significantly higher (*p* = 0.005 and *p* = 0.015 respectively) as compared to group “mild.” Furthermore the use of ACE inhibitors was more frequent in patients with severe outcome (*p* = 0.035). Additionally, in patients of group “severe” the PaO2/FiO2 (ratio) resulted significantly lower (*p* < 0.001), as expected, as well as lymphocytes (*p* < 0.001), while WBC (*p* = 0.033), neutrophils (*p* = 0.004), LDH (*p* = 0.001), CRP (*p* = 0.018), ferritin (*p* < 0.001) and fibrinogen (*p* = 0.013) were all significantly higher than patients with mild symptoms (Table 1). Considering the clinical evolution, the intensive care admission (*p* < 0.001), the proportion of patients requiring ventilation (*p* < 0.001) and the number of deaths (*p* = 0.013) were significantly higher in “severe” group (Table 1).

**Table 1:** Demographic and clinical characteristics (i.e. comorbidities, pharmacological treatments and haematochemical test results) of COVID-19 patients evaluated at their hospitalization.

Variable	Category	All patients	Group A	Group B	*p*-value
		(*N*=97)	(*N*=47)	(*N*=50)	
*Demographic*					
Age (years)	Mean ± SD	60.2 ± 15.7	57.2 ± 18.4	63.0 ± 12.1	0.070*
	Range	22–91	22–91	37–88	
Gender – *N*(%)	Females	32 (33.0)	19 (40.4)	13 (26.0)	0.195^#^
	Males	65 (67.0)	28 (59.6)	37 (74.0)	
*Comorbidities*					
Pneumonia – *N*(%)	No	7 (7.2)	7 (14.9)	0 (0.0)	0.005^#^
	Yes	90 (92.8)	40 (85.1)	50 (100.0)	
	Unknown	0 (0.0)	0 (0.0)	0 (0.0)	
Obesity – *N*(%)	No	66 (68.0)	35 (74.5)	31 (62.0)	0.484^#^
	Yes	23 (23.7)	9 (19.1)	14 (28.0)	
	Unknown	8 (8.2)	3 (6.4)	5 (10.0)	
COPD – *N*(%)	No	87 (89.7)	45 (95.7)	42 (84.0)	0.088^#^
	Yes	6 (6.2)	2 (4.3)	4 (8.0)	
	Unknown	4 (4.1)	0 (0.0)	4 (8.0)	
T2D – *N*(%)	No	76 (78.4)	36 (76.6)	40 (80.0)	0.618^#^
	Yes	20 (20.6)	11 (23.4)	9 (18.0)	
	Unknown	1 (1.0)	0 (0.0)	1 (2.0)	
Hypertension – *N*(%)	No	49 (50.5)	30 (63.8)	19 (38.0)	0.015^#^
	Yes	47 (48.5)	17 (36.2)	30 (60.0)	
	Unknown	1 (1.0)	0 (0.0)	1 (2.0)	
Renal failure – *N*(%)	No	92 (94.8)	43 (91.5)	49 (98.0)	0.051^#^
	Yes	4 (4.1)	4 (8.5)	0 (0.0)	
	Unknown	1 (1.0)	0 (0.0)	1 (2.0)	
Malignancy – *N*(%)	No	91 (93.8)	45 (95.7)	46 (92.0)	0.555^#^
	Yes	4 (4.1)	2 (4.3)	2 (4.0)	
	Unknown	2 (2.1)	0 (0.0)	2 (4.0)	
CVD – *N*(%)	No	71 (73.2)	37 (78.7)	34 (68.0)	0.302^#^
	Yes	25 (25.8)	10 (21.3)	15 (30.0)	
	Unknown	1 (1.0)	0 (0.0)	1 (2.0)	
Neurological diseases – *N*(%)	No	85 (87.6)	39 (83.0)	46 (92.0)	0.114^#^
	Yes	11 (11.3)	8 (17.0)	3 (6.0)	
	Unknown	1 (1.0)	0 (0.0)	1 (2.0)	
Liver disease – *N*(%)	No	87 (89.7)	44 (93.6)	43 (86.0)	0.488^#^
	Yes	9 (9.3)	3 (6.4)	6 (12.0)	
	Unknown	1 (1.0)	0 (0.0)	1 (2.0)	
*Pharmacological treatments (before hospitalization)*					
Drug treatments – *N*(%)	No	23 (23.7)	12 (25.5)	11 (22.0)	0.154^#^
	Yes	58 (59.8)	24 (51.1)	34 (68.0)	
	Unknown	16 (16.5)	11 (23.4)	5 (10.0)	
ACE inhibitors – *N*(%)	No	65 (67.0)	32 (68.1)	33 (66.0)	0.035^#^
	Yes	14 (14.4)	3 (6.4)	11 (22.0)	
	Unknown	18 (18.6)	12 (25.5)	6 (12.0)	
	Yes	4 (4.1)	2 (4.3)	2 (4.0)	
	Unknown	16 (16.5)	11 (23.4)	5 (10.0)	
					
Antibiotics – *N*(%)	No	65 (67.0)	27 (57.4)	38 (76.0)	0.139^#^
	Yes	16 (16.5)	9 (19.1)	7 (14.0)	
	Unknown	16 (16.5)	11 (23.4)	5 (10.0)	
*Haematochemical Blood test*					
PaO2/FiO2 (ratio)	Mean ± SD	299.1 ± 111.2	372.4 ± 87.1	230.1 ± 84.0	<0.001*
	Range	110–524	193–524	110–405	
White blood cell counts (cells/mm3)	Median [IQR]	5.8 [4.8–8.6]	5.4 [4.8–6.7]	7.5 [5.0–9.2]	0.033°
	Range	2.2–27.9	2.6–27.9	2.2–19.4	
Neutrophils (cells/mm3)	Median [IQR]	4.0 [3.0–6.5]	3.6 [2.8–4.6]	5.5 [3.5–7.9]	0.004°
	Range	1.3–26.1	1.4–26.1	1.3–17.2	
Lymphocytes (cells/mm3)	Median [IQR]	1.0 [0.6–1.5]	1.2 [0.9–1.7]	0.8 [0.5–1.1]	<0.001°
	Range	0.1–5.7	0.5–3.2	0.1–5.7	
Hb (g/dl)	Mean ± SD	13.7 ± 1.8	13.4 ± 2.2	14.1 ± 1.3	0.085*
	Range	5.5––17.6	5.5–17.6	11.4–16.6	
LDH (U/L)	Median [IQR]	262.0 [213.0–325.0]	226.5 [189.0–278.2]	294.0 [243.5–383.5]	0.001°
	Range	89–889	89–847	175–889	
CRP (mg/dl)	Median [IQR]	3.7 [1.7–9.1]	2.6 [1.4–5.6]	5.2 [2.4–13.9]	0.018°
	Range	0.0–30.5	0.1–19.6	0.0–30.5	
Ferritin (ng/ml)	Median [IQR]	382.5 [215.2–863.5]	270.0 [171.0–388.0]	589.0 [343.5–1268.5]	<0.001°
	Range	Oct-90	10–1610	15–5390	
Fibrinogen (mg/dl)	Mean ± SD	604.2 ± 218.3	549.5 ± 195.8	664.1 ± 228.1	0.013*
	Range	206–1328	206–1111	265–1328	
D-dimer (ng/ml)	Median [IQR]	661.5 [437.0–993.5]	598.5 [421.5–988.5]	737.5 [490.2–984.5]	0.376°
	Range	144–42885	148–42885	144–30145	
IL-6	Median [IQR]	13.7 [5.3–31.8]	13.1 [6.5–31.2]	16.6 [3.9–44.4]	0.738°
	Range	1.7–507.9	3.7–46.0	1.7–507.9	
*Hospitalization (Clinical) Outcomes*					
Intensive care unit admission – *N*(%)	No	77 (79.4)	47 (100.0)	30 (60.0)	<0.001^#^
	Yes	20 (20.6)	0 (0.0)	20 (40.0)	
Required ventilation – *N*(%) Ventilatory support required	No	47 (48.5)	47 (100.0)	0 (0.0)	<0.001^#^
	NIV	43 (44.3)	0 (0.0)	43 (86.0)	
	IOT	1 (1.0)	0 (0.0)	1 (2.0)	
	IOT + NIV	6 (6.2)	0 (0.0)	6 (12.0)	
Final outcome – N(%)	Discharged	81 (83.5)	43 (91.5)	38 (76.0)	0.013^#^
	Death	8 (8.2)	0 (0.0)	8 (16.0)	
	Transferred	8 (8.2)	4 (8.5)	4 (8.0)	

SD, Standard deviation; IQR, Interquartile Range (i.e. first-third quartiles); Range, Min-max range. Mild: Patients affected by coronavirus disease (COVID-19) with mild symptoms (i.e. low-grade fever, dry cough, fatigue, headache, new loss of taste or smell, gastrointestinal upset, itchy, painful patches on skin); Severe: Patients affected by coronavirus disease (COVID-19) with moderate or severe/critical symptoms (i.e. high-grade fever, chills, deep cough, fatigue and body aches, muscle pain, shortness of breath, chest discomfort, confusion/unresponsiveness, trouble staying awake, eye problems, bluish face/lips). COPD, Chronic obstructive pulmonary disease. T2D, Type 2 diabetes. CVD, Cardiovascular disease. ACE, Angiotensin-converting enzyme. ARB, Angiotensin receptor blockers. NSAIDs, Non-steroidal anti-inflammatory drugs. Hb, Hemoglobin. LDH, lactate dehydrogenase. CRP, C-reactive protein. IL-6, Interleukin 6. Results are reported for all patients and are also stratified according to COVID-19 evolution. ^*^p-value from two-sample test; °*p*-value from Mann–Whitney *U* test; ^#^*p*-value from Fisher exact test.

*Comparison of gut microbiota composition between patients with mild or severe disease evolution.*

**Figure 1:**
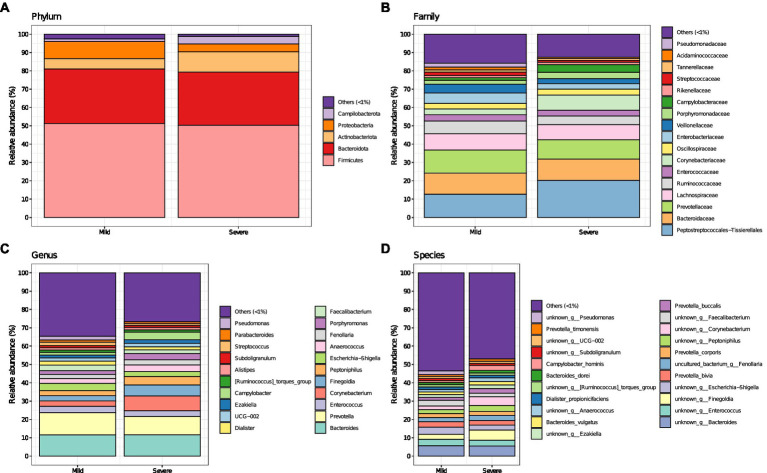
Fecal microbiota composition (i.e., mean relative abundance %) at Phylum **(A)**, Family **(B)**, Genus **(C)**, and Species **(D)** levels in patients with coronavirus disease (COVID-19) at their hospitalization, for mild and severe symptoms separately. “Others (<1%)” category includes bacteria with mean relative abundance less than 1%.

While no changes in Chao1 and Shannon index were observed between group “mild” and group “severe” (Supplementary Figure 1), the different fecal microbiota composition of patients with mild and severe outcome of COVID-19 are reported in Figure 1, with major changes displayed at lower taxonomic levels. Worth of note, at phylum level, Campylobacterota and Actinobacteriota were increased in patients with the worse outcome (group “severe”). The phylum of Campylobacterota includes several pathogens causing diarrhea, cramps, fever and pain. At family level, Peptostreptococcale, Corynebacteriaceae and Campylobacteriaceae increased in group “severe,” while Ruminoccoccaceae (involved in producing short-chain fatty acids) and Enterobacteriaceae decreased. Conversely, in patients with mild disease, Enterobacteriaceae, Veillonellaceae and Streptococcaceae increased. Furthermore, at genus and species level an increase of *Corynebacterium*, *Campylobacter* and *Finegoldia* in group “severe” and *Escherichia-Shigella* and *Faecalibacterium* in group “mild” was observed. PCoA analysis of gut microbiota based on Bray–Curtis dissimilarity (Supplementary Figure 2) plots the difference in the community of fecal microbiota across the two groups (mild and severe) while a rarefaction curve is reported in Supplementary Figure 3.

### PELORA algorithm identified bacterial populations associated to disease evolution

Taking advanteage of the PELORA algorithm we identified clusters of bacterial populations that better discriminate between patients with mild from those with severe COVID-19 evolution based on the relative abundances generated by taxonomic analyses. Table 2 reports the list of the bacteria detected by the algorithm within each cluster. Although every taxon reported concurred to the distinctive cluster which better discriminate patients with mild or severe respiratory failure, we have observed that at Phylum level Proteobacteria, Desulfobacterota, Patescibacteria, Spirochaetota in cluster 1 discriminate the outcome between the two groups and that at Family level Enterobacteriaceae, Elusimicrobiaceae, Sedimentibacteraceae, Atopobiaceae, Sphingomonadaceae, Acidaminococcaceae, Spirochaetaceae, Lachnospiraceae had a greater discriminatory power. At Genus level an increase of *Escherichia-Shigella, Megasphaera, Succiniclasticum, Clostridium_sensu_stricto_13, Sphingomonas, Parvimonas, Candidatus_Stoquefichus, Alloscardovia, Succinivibrio, Klebsiella, Gordonibacter, Proteiniphilum, Catonella, Acinetobacter, Flavonifractor, Gardnerella, Halomonas, Lachnospiraceae_UCG-001, Lachnospiraceae_UCG-003, uncultured_f__Desulfovibrionaceae, uncultured_f__Prevotellaceae, unknown_f__Hungateiclostridiaceae* was observed in cluster 1 and an increase of *Finegoldia, [Clostridium]_methylpentosum_group, Facklamia, Butyrivibrio, Prevotellaceae_NK3B31_group, Corynebacterium, Prevotellaceae_UCG-001, Hymenobacter, Lactobacillus, Actinobaculum Eubacterium, Cutibacterium, Holdemania, Pseudoflavonifractor, Epulopiscium, Saccharimonadales, unknown_f__Corynebacteriaceae* was observed in cluster 2.

**Table 2:** Results from PEnalized LOgistic Regression Analysis (PELORA).

Taxa level	Cluster number	Selected bacteria (within each cluster)	Quantity	Statistics	Mild (*N* = 47)	Severe (*N* = 50)	*p*-value^#^
Phylum	1	Proteobacteria	Relative abundance (%)	Mean ± SD	9.396 ± 17.603	4.291 ± 9.160	–
				Median [IQR]	3.770 [1.058–9.123]	0.794 [0.310–3.511]	
			*Z*-score	Mean ± SD	0.342 ± 0.870	−0.322 ± 1.015	0.001
		Desulfobacterota	Relative abundance (%)	Mean ± SD	0.333 ± 0.489	0.217 ± 0.340	–
				Median [IQR]	0.154 [0.049–0.448]	0.106 [0.001–0.293]	
			*Z*-score°	Mean ± SD	0.149 ± 0.934	−0.140 ± 1.049	0.155
		Patescibacteria	Relative abundance (%)	Mean ± SD	0.022 ± 0.050	0.028 ± 0.161	–
				Median [IQR]	0.001 [0.000–0.018]	0.000 [0.000–0.005]	
			*Z*-score°	Mean ± SD	0.138 ± 1.090	−0.129 ± 0.899	0.190
		Spirochaetota	Relative abundance (%)	Mean ± SD	0.010 ± 0.045	0.001 ± 0.004	–
				Median [IQR]	0.000 [0.000–0.000]	0.000 [0.000–0.000]	
			*Z*-score°	Mean ± SD	0.122 ± 1.255	−0.115 ± 0.673	0.246
		Unassigned	Relative abundance (%)	Mean ± SD	0.017 ± 0.059	0.003 ± 0.008	–
				Median [IQR]	0.003 [0.000–0.008]	0.000 [0.000–0.003]	
			*Z*-score°	Mean ± SD	0.279 ± 1.081	−0.263 ± 0.847	0.007
		Others	Relative abundance (%)	Mean ± SD	0.004 ± 0.006	0.002 ± 0.008	–
				Median [IQR]	0.000 [0.000–0.004]	0.000 [0.000–0.000]	
			*Z*-score°	Mean ± SD	0.229 ± 1.087	−0.215 ± 0.868	0.028
		Cluster centroid	*Z*-score (means)	Mean ± SD	0.210 ± 0.425	−0.197 ± 0.362	<0.001
Family	1	Enterobacteriaceae	Relative abundance (%)	Mean ± SD	5.636 (11.290)	2.952 (8.607)	–
				Median [IQR]	1.112 [0.143, 5.604]	0.171 [0.012, 1.091]	
			*Z*-score°	Mean ± SD	0.314 (0.862)	−0.295 (1.038)	0.002
		Elusimicrobiaceae	Relative abundance (%)	Mean ± SD	0.042 (0.198)	Absent	–
				Median [IQR]	0.000 [0.000, 0.000]		
			*Z*-score°	Mean ± SD	0.154 (1.428)	−0.144 (0.000)	0.143^§^
		Sedimentibacteraceae	Relative abundance (%)	Mean ± SD	0.015 (0.102)	Absent	–
				Median [IQR]	0.000 [0.000, 0.000]		
			*Z*-score°	Mean ± SD	0.108 (1.437)	−0.102 (0.000)	0.302^§^
		Atopobiaceae	Relative abundance (%)	Mean ± SD	0.170 (0.296)	0.203 (1.127)	–
				Median [IQR]	0.023 [0.000, 0.215]	0.004 [0.000, 0.020]	
			*Z*-score°	Mean ± SD	0.200 (1.083)	−0.188 (0.885)	0.056
		Sphingomonadaceae	Relative abundance (%)	Mean ± SD	0.044 (0.106)	0.027 (0.058)	–
				Median [IQR]	0.008 [0.002, 0.036]	0.007 [0.002, 0.019]	
			*Z*-score°	Mean ± SD	0.094 (1.020)	−0.088 (0.983)	0.373
		Acidaminococcaceae	Relative abundance (%)	Mean ± SD	1.547 (2.652)	0.734 (1.696)	–
				Median [IQR]	0.836 [0.042, 1.567]	0.086 [0.005, 0.762]	
			*Z*-score°	Mean ± SD	0.204 (1.002)	−0.192 (0.969)	0.051
		Spirochaetaceae	Relative abundance (%)	Mean ± SD	0.010 (0.045)	0.001 (0.004)	–
				Median [IQR]	0.000 [0.000, 0.000]	0.000 [0.000, 0.000]	
			*Z*-score°	Mean ± SD	0.122 (1.255)	−0.115 (0.674)	0.246
		Lachnospiraceae	Relative abundance (%)	Mean ± SD	8.918 (8.626)	8.263 (11.364)	–
				Median [IQR]	6.893 [2.324, 12.377]	4.556 [1.478, 9.537]	
			*Z*-score°	Mean ± SD	0.113 (0.880)	−0.106 (1.100)	0.283
		Unassigned	Relative abundance (%)	Mean ± SD	0.017 (0.059)	0.003 (0.008)	–
				Median [IQR]	0.003 [0.000, 0.008]	0.000 [0.000, 0.003]	
			*Z*-score°	Mean ± SD	0.279 (1.081)	−0.263 (0.847)	0.007
		Cluster centroid	*Z*-score (means)	Mean ± SD	0.176 (0.352)	−0.166 (0.243)	<0.001
Genus	1	Escherichia-Shigella	Relative abundance (%)	Mean ± SD	3.875 (7.121)	2.742 (8.509)	–
				Median [IQR]	0.677 [0.100, 3.813]	0.120 [0.007, 0.960]	
			*Z*-score°	Mean ± SD	0.323 (0.810)	−0.304 (1.072)	0.002
		Megasphaera	Relative abundance (%)	Mean ± SD	0.700 (1.627)	0.147 (0.554)	–
				Median [IQR]	0.008 [0.000, 0.242]	0.000 [0.000, 0.003]	
			*Z*-score°	Mean ± SD	0.305 (1.092)	−0.286 (0.817)	0.003
		Succiniclasticum	Relative abundance (%)	Mean ± SD	0.047 (0.218)	0.003 (0.010)	–
				Median [IQR]	0.000 [0.000, 0.001]	0.000 [0.000, 0.000]	
			*Z*-score°	Mean ± SD	0.240 (1.237)	−0.226 (0.644)	0.021
		Clostridium_sensu_stricto_13	Relative abundance (%)	Mean ± SD	0.046 (0.217)	0.001 (0.003)	–
				Median [IQR]	0.000 [0.000, 0.000]	0.000 [0.000, 0.000]	
			*Z*-score°	Mean ± SD	0.125 (1.335)	−0.118 (0.507)	0.234
		Sphingomonas	Relative abundance (%)	Mean ± SD	0.041 (0.101)	0.024 (0.055)	–
				Median [IQR]	0.008 [0.002, 0.036]	0.007 [0.002, 0.019]	
			*Z*-score°	Mean ± SD	0.099 (1.016)	−0.093 (0.986)	0.347
		Parvimonas	Relative abundance (%)	Mean ± SD	0.195 (0.853)	0.023 (0.087)	–
				Median [IQR]	0.000 [0.000, 0.004]	0.000 [0.000, 0.000]	
			*Z*-score°	Mean ± SD	0.165 (1.135)	−0.155 (0.836)	0.116
		Candidatus_Stoquefichus	Relative abundance (%)	Mean ± SD	0.011 (0.072)	Absent	–
				Median [IQR]	0.000 [0.000, 0.000]		
			*Z*-score°	Mean ± SD	0.108 (1.437)	−0.102 (0.000)	0.302^§^
		Alloscardovia	Relative abundance (%)	Mean ± SD	0.010 (0.064)	Absent	–
				Median [IQR]	0.000 [0.000, 0.000]		
			*Z*-score°	Mean ± SD	0.176 (1.423)	−0.166 (0.000)	0.036^§^
		Succinivibrio	Relative abundance (%)	Mean ± SD	0.134 (0.882)	0.001 (0.005)	–
				Median [IQR]	0.000 [0.000, 0.000]	0.000 [0.000, 0.000]	
			*Z*-score°	Mean ± SD	0.134 (1.330)	−0.126 (0.515)	0.204
		Klebsiella	Relative abundance (%)	Mean ± SD	1.375 (7.902)	0.025 (0.073)	–
				Median [IQR]	0.000 [0.000, 0.016]	0.000 [0.000, 0.009]	
			*Z*-score°	Mean ± SD	0.173 (1.134)	−0.163 (0.834)	0.098
		Gordonibacter	Relative abundance (%)	Mean ± SD	0.009 (0.035)	0.003 (0.009)	–
				Median [IQR]	0.000 [0.000, 0.001]	0.000 [0.000, 0.000]	
			*Z*-score°	Mean ± SD	0.073 (1.113)	−0.069 (0.886)	0.486
		Proteiniphilum	Relative abundance (%)	Mean ± SD	0.039 (0.265)	Absent	–
				Median [IQR]	0.000 [0.000, 0.000]		
			*Z*-score°	Mean ± SD	0.108 (1.437)	−0.102 (0.000)	0.302^§^
		Catonella	Relative abundance (%)	Mean ± SD	0.006 (0.037)	Absent	
				Median [IQR]	0.000 [0.000, 0.000]		
			*Z*-score°	Mean ± SD	0.108 (1.437)	−0.102 (0.000)	0.302^§^
		Acinetobacter	Relative abundance (%)	Mean ± SD	0.034 (0.222)	0.003 (0.009)	–
				Median [IQR]	0.000 [0.000, 0.000]	0.000 [0.000, 0.000]	
			*Z*-score°	Mean ± SD	0.063 (1.118)	−0.059 (0.882)	0.553
		Flavonifractor	Relative abundance (%)	Mean ± SD	0.103 (0.145)	0.092 (0.227)	–
				Median [IQR]	0.042 [0.002, 0.139]	0.013 [0.000, 0.084]	
			*Z*-score°	Mean ± SD	0.132 (0.989)	−0.124 (1.004)	0.209
		Gardnerella	Relative abundance (%)	Mean ± SD	0.028 (0.149)	0.001 (0.003)	–
				Median [IQR]	0.000 [0.000, 0.000]	0.000 [0.000, 0.000]	
			*Z*-score°	Mean ± SD	0.161 (1.316)	−0.152 (0.533)	0.124
		W5053	Relative abundance (%)	Mean ± SD	0.026 (0.079)	0.015 (0.058)	–
				Median [IQR]	0.000 [0.000, 0.002]	0.000 [0.000, 0.000]	
			*Z*-score°	Mean ± SD	0.135 (1.120)	−0.126 (0.865)	0.2
		Halomonas	Relative abundance (%)	Mean ± SD	0.010 (0.027)	0.008 (0.018)	–
				Median [IQR]	0.000 [0.000, 0.007]	0.001 [0.000, 0.006]	
			*Z*-score°	Mean ± SD	−0.010 (1.042)	0.010 (0.970)	0.921
		Lachnospiraceae_UCG-001	Relative abundance (%)	Mean ± SD	0.018 (0.049)	0.006 (0.025)	–
				Median [IQR]	0.000 [0.000, 0.001]	0.000 [0.000, 0.000]	
			*Z*-score°	Mean ± SD	0.199 (1.188)	−0.187 (0.749)	0.057
		Lachnospiraceae_UCG-003	Relative abundance (%)	Mean ± SD	0.014 (0.059)	0.047 (0.330)	–
				Median [IQR]	0.000 [0.000, 0.000]	0.000 [0.000, 0.000]	
			*Z*-score°	Mean ± SD	0.104 (1.119)	−0.098 (0.874)	0.322
		uncultured_f__Desulfovibrionaceae	Relative abundance (%)	Mean ± SD	0.017 (0.054)	0.008 (0.022)	–
				Median [IQR]	0.000 [0.000, 0.007]	0.000 [0.000, 0.003]	
			*Z*-score°	Mean ± SD	0.097 (1.049)	−0.091 (0.954)	0.358
		uncultured_f__Prevotellaceae	Relative abundance (%)	Mean ± SD	0.029 (0.105)	0.004 (0.010)	–
				Median [IQR]	0.000 [0.000, 0.000]	0.000 [0.000, 0.000]	
			*Z*-score°	Mean ± SD	0.055 (1.152)	−0.052 (0.841)	0.6
		unknown_f__Hungateiclostridiaceae	Relative abundance (%)	Mean ± SD	0.011 (0.040)	0.001 (0.006)	–
				Median [IQR]	0.000 [0.000, 0.000]	0.000 [0.000, 0.000]	
			*Z*-score°	Mean ± SD	0.203 (1.258)	−0.191 (0.630)	0.052
		Unassigned	Relative abundance (%)	Mean ± SD	0.017 (0.059)	0.003 (0.008)	–
				Median [IQR]	0.003 [0.000, 0.008]	0.000 [0.000, 0.003]	
			*Z*-score°	Mean ± SD	0.279 (1.081)	−0.263 (0.847)	0.007
		Cluster centroid	*Z*-score (means)	Mean ± SD	0.148 (0.184)	−0.139 (0.133)	<0.001
Genus	2	Finegoldia	Relative abundance (%)	Mean ± SD	2.855 (4.707)	6.009 (8.986)	–
				Median [IQR]	1.077 [0.060, 4.050]	2.613 [0.594, 8.657]	
			*Z*-score°	Mean ± SD	−0.308 (1.149)	0.290 (0.737)	0.003
		[Clostridium]_methylpentosum_group	Relative abundance (%)	Mean ± SD	0.029 (0.111)	0.008 (0.015)	–
				Median [IQR]	0.000 [0.000, 0.001]	0.002 [0.000, 0.008]	
			*Z*-score°	Mean ± SD	−0.158 (1.062)	0.149 (0.924)	0.131
		Facklamia	Relative abundance (%)	Mean ± SD	0.053 (0.162)	0.263 (0.848)	–
				Median [IQR]	0.000 [0.000, 0.013]	0.010 [0.001, 0.057]	
			*Z*-score°	Mean ± SD	−0.284 (0.915)	0.267 (1.012)	0.006
		Butyrivibrio	Relative abundance (%)	Mean ± SD	0.000 (0.001)	0.015 (0.072)	–
				Median [IQR]	0.000 [0.000, 0.000]	0.000 [0.000, 0.000]	
			*Z*-score°	Mean ± SD	−0.165 (0.376)	0.155 (1.332)	0.115
		Prevotellaceae_NK3B31_group	Relative abundance (%)	Mean ± SD	0.110 (0.478)	0.299 (0.814)	–
				Median [IQR]	0.000 [0.000, 0.000]	0.000 [0.000, 0.010]	
			*Z*-score°	Mean ± SD	−0.173 (0.849)	0.163 (1.108)	0.098
		Corynebacterium	Relative abundance (%)	Mean ± SD	2.962 (8.656)	7.991 (19.841)	–
				Median [IQR]	0.403 [0.037, 1.567]	1.470 [0.307, 4.392]	
			*Z*-score°	Mean ± SD	−0.291 (1.011)	0.273 (0.917)	0.005
		GCA-900066575	Relative abundance (%)	Mean ± SD	0.026 (0.049)	0.029 (0.057)	–
				Median [IQR]	0.001 [0.000, 0.022]	0.009 [0.000, 0.029]	
			*Z*-score°	Mean ± SD	−0.106 (1.009)	0.100 (0.991)	0.313
		Prevotellaceae_UCG-001	Relative abundance (%)	Mean ± SD	0.001 (0.002)	0.039 (0.164)	–
				Median [IQR]	0.000 [0.000, 0.000]	0.000 [0.000, 0.000]	
			*Z*-score°	Mean ± SD	−0.167 (0.396)	0.157 (1.327)	0.111
		Hymenobacter	Relative abundance (%)	Mean ± SD	Absent	0.003 (0.021)	–
				Median [IQR]		0.000 [0.000, 0.000]	
			*Z*-score°	Mean ± SD	−0.102 (0.000)	0.095 (1.393)	0.332^§^
		Lactobacillus	Relative abundance (%)	Mean ± SD	0.175 (0.649)	0.193 (0.827)	–
				Median [IQR]	0.004 [0.000, 0.038]	0.003 [0.000, 0.027]	
			*Z*-score°	Mean ± SD	0.013 (1.027)	−0.013 (0.985)	0.899
		Actinobaculum	Relative abundance (%)	Mean ± SD	Absent	0.009 (0.046)	–
				Median [IQR]		0.000 [0.000, 0.000]	
			*Z*-score°	Mean ± SD	−0.217 (0.000)	0.204 (1.368)	0.027^§^
		Eubacterium	Relative abundance (%)	Mean ± SD	0.008 (0.024)	0.012 (0.038)	–
				Median [IQR]	0.000 [0.000, 0.001]	0.000 [0.000, 0.000]	
			*Z*-score°	Mean ± SD	0.023 (0.979)	−0.022 (1.029)	0.827
		Cutibacterium	Relative abundance (%)	Mean ± SD	0.015 (0.044)	0.021 (0.089)	–
				Median [IQR]	0.000 [0.000, 0.002]	0.000 [0.000, 0.006]	
			*Z*-score°	Mean ± SD	−0.070 (1.011)	0.066 (0.996)	0.504
		Holdemania	Relative abundance (%)	Mean ± SD	0.009 (0.017)	0.025 (0.063)	–
				Median [IQR]	0.000 [0.000, 0.011]	0.004 [0.000, 0.019]	
			*Z*-score°	Mean ± SD	−0.174 (0.921)	0.164 (1.052)	0.096
		Pseudoflavonifractor	Relative abundance (%)	Mean ± SD	0.002 (0.008)	0.009 (0.044)	–
				Median [IQR]	0.000 [0.000, 0.000]	0.000 [0.000, 0.000]	
			*Z*-score°	Mean ± SD	−0.105 (0.785)	0.099 (1.166)	0.318
		Epulopiscium	Relative abundance (%)	Mean ± SD	0.008 (0.050)	0.000 (0.001)	–
				Median [IQR]	0.000 [0.000, 0.000]	0.000 [0.000, 0.000]	
			*Z*-score°	Mean ± SD	0.055 (1.306)	−0.052 (0.593)	0.602
		Saccharimonadales	Relative abundance (%)	Mean ± SD	0.010 (0.036)	0.024 (0.161)	–
				Median [IQR]	0.000 [0.000, 0.000]	0.000 [0.000, 0.000]	
			*Z*-score°	Mean ± SD	0.086 (1.104)	−0.081 (0.895)	0.415
		unknown_f__Corynebacteriaceae	Relative abundance (%)	Mean ± SD	Absent	0.008 (0.049)	–
				Median [IQR]		0.000 [0.000, 0.000]	
			*Z*-score°	Mean ± SD	−0.143 (0.000)	0.134 (1.386)	0.168^§^
		Cluster centroid	*Z*-score (means)	Mean ± SD	−0.127 (0.185)	0.119 (0.197)	<0.001
Species	1	Actinobaculum_massiliense	Relative abundance (%)	Mean ± SD	Absent	0.009 (0.046)	–
				Median [IQR]		0.000 [0.000, 0.000]	
			*Z*-score°	Mean ± SD	−0.217 (0.000)	0.204 (1.368)	0.027^§^
		Anaerococcus_hydrogenalis	Relative abundance (%)	Mean ± SD	0.003 (0.021)	0.001 (0.003)	–
				Median [IQR]	0.000 [0.000, 0.000]	0.000 [0.000, 0.000]	
			*Z*-score°	Mean ± SD	−0.061 (1.021)	0.057 (0.987)	0.565
		bacterium_NLAE-zl-H60	Relative abundance (%)	Mean ± SD	0.000 (0.000)	0.033 (0.196)	–
				Median [IQR]	0.000 [0.000, 0.000]	0.000 [0.000, 0.000]	
			*Z*-score°	Mean ± SD	−0.211 (0.364)	0.198 (1.324)	0.044
		Brevibacterium_ravenspurgense	Relative abundance (%)	Mean ± SD	0.002 (0.007)	0.060 (0.324)	–
				Median [IQR]	0.000 [0.000, 0.000]	0.000 [0.000, 0.000]	
			*Z*-score°	Mean ± SD	−0.130 (0.607)	0.122 (1.258)	0.218
		Corynebacterium_appendicis	Relative abundance (%)	Mean ± SD	0.007 (0.043)	0.107 (0.671)	–
				Median [IQR]	0.000 [0.000, 0.000]	0.000 [0.000, 0.006]	
			*Z*-score°	Mean ± SD	−0.261 (0.661)	0.246 (1.193)	0.012
		Corynebacterium_frankenforstense	Relative abundance (%)	Mean ± SD	0.000 (0.001)	0.005 (0.029)	–
				Median [IQR]	0.000 [0.000, 0.000]	0.000 [0.000, 0.000]	
			*Z*-score°	Mean ± SD	−0.240 (0.374)	0.226 (1.312)	0.021
		Cutibacterium_granulosum	Relative abundance (%)	Mean ± SD	0.000 (0.000)	0.003 (0.018)	–
				Median [IQR]	0.000 [0.000, 0.000]	0.000 [0.000, 0.000]	
			*Z*-score°	Mean ± SD	−0.179 (0.179)	0.168 (1.367)	0.088
		Facklamia_languida	Relative abundance (%)	Mean ± SD	0.006 (0.033)	0.048 (0.171)	–
				Median [IQR]	0.000 [0.000, 0.000]	0.000 [0.000, 0.006]	
			*Z*-score°	Mean ± SD	−0.346 (0.646)	0.325 (1.160)	0.001
		Faecalitalea_cylindroides	Relative abundance (%)	Mean ± SD	0.005 (0.027)	0.005 (0.021)	–
				Median [IQR]	0.000 [0.000, 0.000]	0.000 [0.000, 0.000]	
			*Z*-score°	Mean ± SD	−0.007 (0.990)	0.007 (1.019)	0.947
		gut_metagenome_o__Bacteroidales	Relative abundance (%)	Mean ± SD	0.000 (0.000)	0.014 (0.067)	–
				Median [IQR]	0.000 [0.000, 0.000]	0.000 [0.000, 0.000]	
			*Z*-score°	Mean ± SD	−0.167 (0.113)	0.157 (1.377)	0.111
		Haemophilus_pittmaniae	Relative abundance (%)	Mean ± SD	Absent	0.003 (0.017)	–
				Median [IQR]		0.000 [0.000, 0.000]	
			*Z*-score°	Mean ± SD	−0.102 (0.000)	0.095 (1.393)	0.332^§^
		Marseilla_massiliensis	Relative abundance (%)	Mean ± SD	Absent	0.033 (0.153)	–
				Median [IQR]		0.000 [0.000, 0.000]	
			*Z*-score°	Mean ± SD	−0.203 (0.000)	0.191 (1.372)	0.049^§^
		Moryella_sp.	Relative abundance (%)	Mean ± SD	Absent	0.005 (0.024)	–
				Median [IQR]		0.000 [0.000, 0.000]	
			*Z*-score°	Mean ± SD	−0.175 (0.000)	0.164 (1.379)	0.090^§^
		Pseudoflavonifractor_sp.	Relative abundance (%)	Mean ± SD	0.000 (0.000)	0.005 (0.029)	–
				Median [IQR]	0.000 [0.000, 0.000]	0.000 [0.000, 0.000]	
			*Z*-score°	Mean ± SD	−0.160 (0.319)	0.151 (1.347)	0.127
		Streptococcus_parasanguinis	Relative abundance (%)	Mean ± SD	0.033 (0.151)	0.015 (0.041)	–
				Median [IQR]	0.000 [0.000, 0.009]	0.000 [0.000, 0.013]	
			*Z*-score°	Mean ± SD	−0.041 (1.034)	0.039 (0.976)	0.694
		uncultured_bacterium_g__[Bacteroides]_pectinophilus_group	Relative abundance (%)	Mean ± SD	Absent	0.003 (0.020)	–
				Median [IQR]		0.000 [0.000, 0.000]	
			*Z*-score°	Mean ± SD	−0.128 (0.000)	0.121 (1.389)	0.168^§^
		uncultured_bacterium_g__Finegoldia	Relative abundance (%)	Mean ± SD	0.049 (0.161)	0.492 (1.906)	–
				Median [IQR]	0.011 [0.000, 0.032]	0.059 [0.006, 0.173]	
			*Z*-score°	Mean ± SD	−0.377 (0.884)	0.354 (0.981)	<0.001
		uncultured_bacterium_g__Helcococcus	Relative abundance (%)	Mean ± SD	0.000 (0.001)	0.028 (0.191)	–
				Median [IQR]	0.000 [0.000, 0.000]	0.000 [0.000, 0.000]	
			*Z*-score°	Mean ± SD	−0.114 (0.445)	0.108 (1.323)	0.277
		uncultured_bacterium_g__Pseudoflavonifractor	Relative abundance (%)	Mean ± SD	0.002 (0.008)	0.009 (0.044)	–
				Median [IQR]	0.000 [0.000, 0.000]	0.000 [0.000, 0.000]	
			*Z*-score°	Mean ± SD	−0.080 (0.792)	0.075 (1.166)	0.446
		uncultured_Clostridiales_g__[Ruminococcus]_torques_group	Relative abundance (%)	Mean ± SD	0.002 (0.007)	0.008 (0.039)	–
				Median [IQR]	0.000 [0.000, 0.000]	0.000 [0.000, 0.000]	
			*Z*-score°	Mean ± SD	−0.125 (0.841)	0.118 (1.125)	0.233
		uncultured_organism_g__Butyrivibrio	Relative abundance (%)	Mean ± SD	Absent	0.010 (0.069)	–
				Median [IQR]		0.000 [0.000, 0.000]	
			*Z*-score°	Mean ± SD	−0.135 (0.000)	0.127 (1.388)	0.168^§^
		unknown_g__Corynebacterium	Relative abundance (%)	Mean ± SD	2.070 (5.971)	4.746 (13.705)	–
				Median [IQR]	0.103 [0.015, 1.114]	0.405 [0.078, 2.399]	
			*Z*-score°	Mean ± SD	−0.266 (1.068)	0.250 (0.870)	0.01
		unknown_g__Cutibacterium	Relative abundance (%)	Mean ± SD	0.003 (0.008)	0.006 (0.025)	–
				Median [IQR]	0.000 [0.000, 0.000]	0.000 [0.000, 0.000]	
			*Z*-score°	Mean ± SD	−0.116 (0.911)	0.109 (1.075)	0.271
		unknown_g__Fenollaria	Relative abundance (%)	Mean ± SD	0.027 (0.085)	0.043 (0.094)	–
				Median [IQR]	0.000 [0.000, 0.002]	0.000 [0.000, 0.027]	
			*Z*-score°	Mean ± SD	−0.139 (0.912)	0.131 (1.069)	0.186
		unknown_g__Holdemania	Relative abundance (%)	Mean ± SD	0.008 (0.017)	0.022 (0.062)	–
				Median [IQR]	0.000 [0.000, 0.004]	0.000 [0.000, 0.014]	
			*Z*-score°	Mean ± SD	−0.173 (0.893)	0.163 (1.074)	0.098
		unknown_g__Intestinimonas	Relative abundance (%)	Mean ± SD	0.017 (0.085)	0.022 (0.060)	–
				Median [IQR]	0.000 [0.000, 0.003]	0.001 [0.000, 0.011]	
			*Z*-score°	Mean ± SD	−0.204 (0.880)	0.192 (1.074)	0.051
		unknown_g__Lactobacillus	Relative abundance (%)	Mean ± SD	0.129 (0.523)	0.190 (0.828)	–
				Median [IQR]	0.000 [0.000, 0.014]	0.001 [0.000, 0.017]	
			*Z*-score°	Mean ± SD	−0.043 (0.989)	0.040 (1.019)	0.686
		unknown_g__Merdibacter	Relative abundance (%)	Mean ± SD	0.000 (0.000)	0.003 (0.015)	–
				Median [IQR]	0.000 [0.000, 0.000]	0.000 [0.000, 0.000]	
			*Z*-score°	Mean ± SD	−0.123 (0.388)	0.116 (1.338)	0.242
		Cluster centroid	*Z*-score (means)	Mean ± SD	−0.162 (0.103)	0.152 (0.124)	<0.001
	2	Absiella_argi	Relative abundance (%)	Mean ± SD	0.000 (0.001)	0.007 (0.049)	–
				Median [IQR]	0.000 [0.000, 0.000]	0.000 [0.000, 0.000]	
			*Z*-score°	Mean ± SD	−0.063 (0.526)	0.059 (1.301)	0.549
		Anaerococcus_hydrogenalis	Relative abundance (%)	Mean ± SD	0.003 (0.021)	0.001 (0.003)	–
				Median [IQR]	0.000 [0.000, 0.000]	0.000 [0.000, 0.000]	
			*Z*-score°	Mean ± SD	−0.061 (1.021)	0.057 (0.987)	0.565
		Bacteroidia_bacterium	Relative abundance (%)	Mean ± SD	0.006 (0.035)	0.001 (0.006)	–
				Median [IQR]	0.000 [0.000, 0.000]	0.000 [0.000, 0.000]	
			*Z*-score°	Mean ± SD	0.076 (1.199)	−0.072 (0.774)	0.468
		Blautia_hydrogenotrophica	Relative abundance (%)	Mean ± SD	0.007 (0.018)	0.015 (0.071)	–
				Median [IQR]	0.000 [0.000, 0.000]	0.000 [0.000, 0.000]	
			*Z*-score°	Mean ± SD	0.045 (1.020)	−0.042 (0.990)	0.673
		Butyricicoccus_pullicaecorum	Relative abundance (%)	Mean ± SD	0.004 (0.023)	0.009 (0.062)	–
				Median [IQR]	0.000 [0.000, 0.000]	0.000 [0.000, 0.000]	
			*Z*-score°	Mean ± SD	0.031 (1.008)	−0.029 (1.002)	0.768
		Candidatus_Saccharibacteria	Relative abundance (%)	Mean ± SD	0.007 (0.033)	0.000 (0.001)	–
				Median [IQR]	0.000 [0.000, 0.000]	0.000 [0.000, 0.000]	
			*Z*-score°	Mean ± SD	0.173 (1.368)	−0.163 (0.381)	0.099
		Corynebacterium_riegelii	Relative abundance (%)	Mean ± SD	0.003 (0.016)	Absent	–
				Median [IQR]	0.000 [0.000, 0.000]		
			*Z*-score°	Mean ± SD	0.183 (1.421)	−0.172 (0.000)	0.071^§^
		Dialister_invisus	Relative abundance (%)	Mean ± SD	0.106 (0.362)	0.099 (0.462)	–
				Median [IQR]	0.000 [0.000, 0.008]	0.000 [0.000, 0.000]	
			*Z*-score°	Mean ± SD	0.223 (1.102)	−0.210 (0.853)	0.032
		Dialister_pneumosintes	Relative abundance (%)	Mean ± SD	0.304 (0.822)	0.035 (0.106)	–
				Median [IQR]	0.000 [0.000, 0.081]	0.000 [0.000, 0.001]	
			*Z*-score°	Mean ± SD	0.249 (1.136)	−0.234 (0.795)	0.017
		Lactobacillus_harbinensis	Relative abundance (%)	Mean ± SD	0.005 (0.035)	Absent	–
				Median [IQR]	0.000 [0.000, 0.000]		
			*Z*-score°	Mean ± SD	0.108 (1.437)	−0.102 (0.000)	0.302^§^
		Lactobacillus_iners	Relative abundance (%)	Mean ± SD	0.014 (0.074)	0.000 (0.000)	–
				Median [IQR]	0.000 [0.000, 0.000]	0.000 [0.000, 0.000]	
			*Z*-score°	Mean ± SD	0.181 (1.421)	−0.170 (0.062)	0.084
		metagenome_g__Prevotellaceae_NK3B31_group	Relative abundance (%)	Mean ± SD	0.003 (0.018)	Absent	–
				Median [IQR]	0.000 [0.000, 0.000]		
			*Z*-score°	Mean ± SD	0.151 (1.429)	−0.142 (0.000)	0.143^§^
		Parabacteroides_gordonii	Relative abundance (%)	Mean ± SD	0.019 (0.127)	0.000 (0.001)	–
				Median [IQR]	0.000 [0.000, 0.000]	0.000 [0.000, 0.000]	
			*Z*-score°	Mean ± SD	0.115 (1.356)	−0.109 (0.456)	0.272
		Sutterellaceae_bacterium	Relative abundance (%)	Mean ± SD	0.088 (0.503)	0.003 (0.018)	–
				Median [IQR]	0.000 [0.000, 0.000]	0.000 [0.000, 0.000]	
			*Z*-score°	Mean ± SD	0.178 (1.279)	−0.167 (0.604)	0.09
		uncultured_bacterium_g__[Eubacterium]_eligens_group	Relative abundance (%)	Mean ± SD	0.006 (0.041)	Absent	–
				Median [IQR]	0.000 [0.000, 0.000]		
			*Z*-score°	Mean ± SD	0.108 (1.437)	−0.102 (0.000)	0.302^§^
		uncultured_bacterium_g__Fusobacterium	Relative abundance (%)	Mean ± SD	0.007 (0.041)	Absent	–
				Median [IQR]	0.000 [0.000, 0.000]		
			*Z*-score°	Mean ± SD	0.219 (1.411)	−0.206 (0.000)	0.019^§^
		uncultured_bacterium_g__TM7x	Relative abundance (%)	Mean ± SD	0.007 (0.019)	0.004 (0.013)	–
				Median [IQR]	0.000 [0.000, 0.007]	0.000 [0.000, 0.001]	
			*Z*-score°	Mean ± SD	0.197 (1.096)	−0.185 (0.871)	0.06
		uncultured_Wautersiella	Relative abundance (%)	Mean ± SD	0.054 (0.347)	0.001 (0.003)	–
				Median [IQR]	0.000 [0.000, 0.000]	0.000 [0.000, 0.000]	
			*Z*-score°	Mean ± SD	0.147 (1.340)	−0.138 (0.484)	0.161
		unidentified_marine	Relative abundance (%)	Mean ± SD	0.040 (0.214)	0.055 (0.344)	–
				Median [IQR]	0.000 [0.000, 0.000]	0.000 [0.000, 0.000]	
			*Z*-score°	Mean ± SD	0.081 (1.058)	−0.076 (0.947)	0.441
		unknown_f__Hungateiclostridiaceae	Relative abundance (%)	Mean ± SD	0.011 (0.040)	0.001 (0.006)	–
				Median [IQR]	0.000 [0.000, 0.000]	0.000 [0.000, 0.000]	
			*Z*-score°	Mean ± SD	0.203 (1.258)	−0.191 (0.630)	0.052
		unknown_g__Candidatus_Stoquefichus	Relative abundance (%)	Mean ± SD	0.011 (0.072)	Absent	–
				Median [IQR]	0.000 [0.000, 0.000]		
			*Z*-score°	Mean ± SD	0.108 (1.437)	−0.102 (0.000)	0.302^§^
		Unknown_g__Clostridia_vadinBB60_Group	Relative abundance (%)	Mean ± SD	0.007 (0.020)	0.005 (0.017)	–
				Median [IQR]	0.000 [0.000, 0.000]	0.000 [0.000, 0.000]	
			*Z*-score°	Mean ± SD	0.048 (1.055)	−0.045 (0.954)	0.65
		unknown_g__Faecalitalea	Relative abundance (%)	Mean ± SD	0.021 (0.139)	0.001 (0.001)	–
				Median [IQR]	0.000 [0.000, 0.000]	0.000 [0.000, 0.000]	
			*Z*-score°	Mean ± SD	0.092 (1.312)	−0.087 (0.572)	0.38
		unknown_g__leptotrichia	Relative abundance (%)	Mean ± SD	0.028 (0.183)	Absent	–
				Median [IQR]	0.000 [0.000, 0.000]		
			*Z*-score°	Mean ± SD	0.147 (1.430)	−0.138 (0.000)	0.143^§^
		unknown_g__negativibacillus	Relative abundance (%)	Mean ± SD	0.010 (0.040)	0.005 (0.025)	–
				Median [IQR]	0.000 [0.000, 0.000]	0.000 [0.000, 0.000]	
			*Z*-score°	Mean ± SD	0.121 (1.142)	−0.113 (0.842)	0.251
		unknown_g__Phascolarctobacterium	Relative abundance (%)	Mean ± SD	0.700 (1.012)	0.467 (0.850)	–
				Median [IQR]	0.324 [0.001, 1.041]	0.044 [0.000, 0.537]	
			*Z*-score°	Mean ± SD	0.085 (1.043)	−0.080 (0.962)	0.42
		unknown_g__Pseudomonas	Relative abundance (%)	Mean ± SD	2.064 (14.100)	0.015 (0.083)	–
				Median [IQR]	0.000 [0.000, 0.006]	0.000 [0.000, 0.005]	
			*Z*-score°	Mean ± SD	0.084 (1.203)	−0.079 (0.767)	0.426
		unknown_g__Sphingomonas	Relative abundance (%)	Mean ± SD	0.041 (0.101)	0.024 (0.055)	–
				Median [IQR]	0.008 [0.002, 0.036]	0.007 [0.002, 0.019]	
			*Z*-score°	Mean ± SD	0.099 (1.016)	−0.093 (0.986)	0.347
		Unassigned	Relative abundance (%)	Mean ± SD	0.017 (0.059)	0.003 (0.008)	–
				Median [IQR]	0.003 [0.000, 0.008]	0.000 [0.000, 0.003]	
			*Z*-score	Mean ± SD	0.279 (1.081)	−0.263 (0.847)	0.007
		Cluster centroid	*Z*-score (means)	Mean ± SD	0.124 (0.109)	−0.117 (0.096)	<0.001

IQR: Interquartile range (i.e., first-third quartiles); SD: Standard Deviation; Absent: all values are 0%. °Standardized *Z*-score: as a first step, the relative abundance (%) of each bacterium was logit transformed (so that values can theoretically range from negative to positive infinity) and, as a second step, the *Z*-score was computed by standardizing the transformed variable (i.e., taking the variable values, subtracting its mean and dividing by its SD). The centroid is calculated as the average of *Z*-scores for all those variables selected within each cluster. ^#^All *p*-values were derived from two-sample t-test on *Z*-scores with the exception of those marked as “^§^” which instead were derived from the nonparametric Mann–Whitney U test. The latter was performed in presence of no variance in one of the two groups (i.e., when the group has all values equals to 0% - denoted as “Absent”). The PELORA algorithm identified clusters of bacterial populations such that the linear combination of their abundances (*Z*-scores) is differential between COVID-19 patients with mild and severe symptoms, respectively.

**Figure 2:**
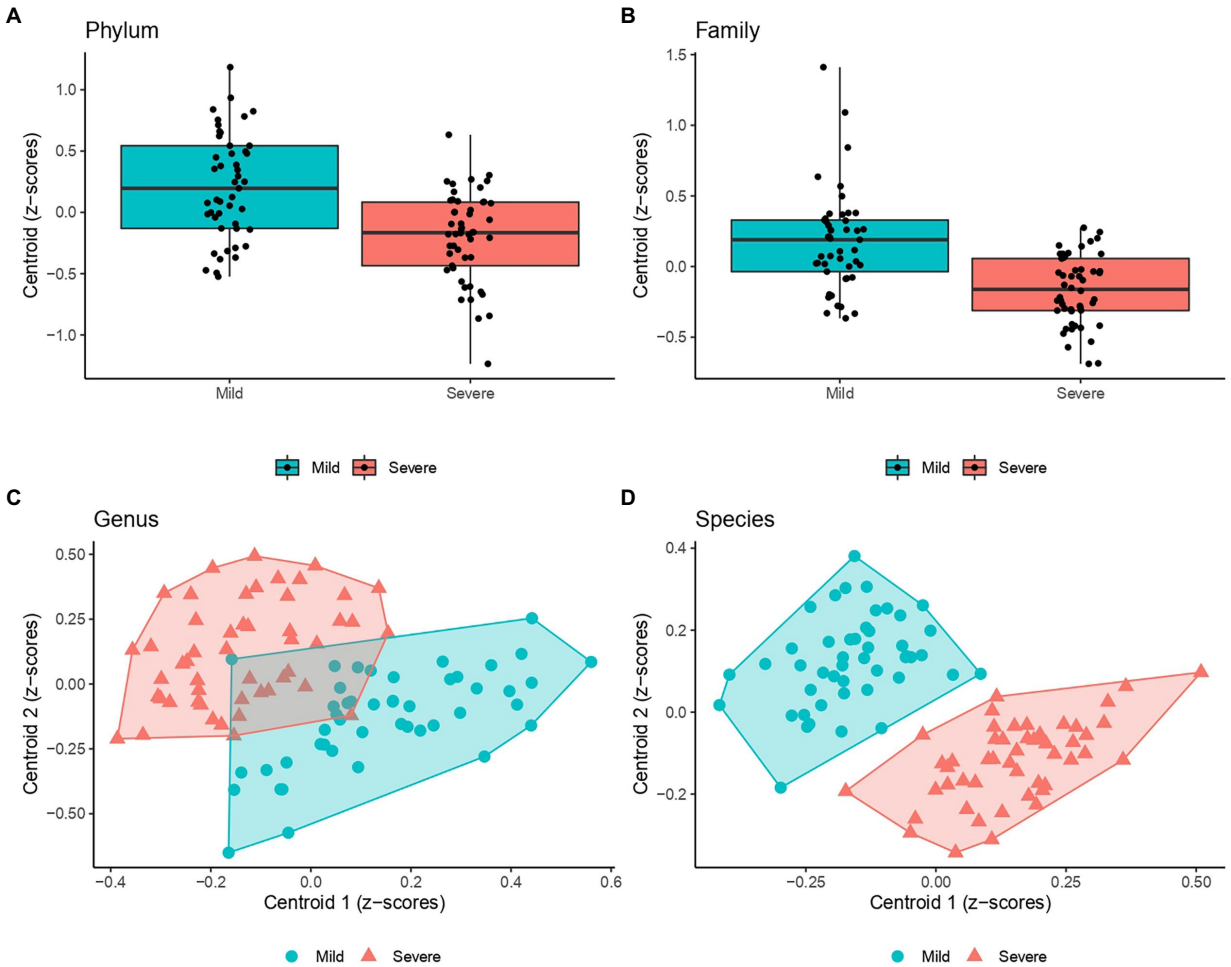
Scatter plots of the *Z*-scores computed within each cluster (i.e., centroid) detected by PEnalized LOgistic Regression Analysis at Genus **(C)**, and Species **(D)**. Each point represents the *Z*-scores pair computed at each individual and were filled with blue and red colors to designate COVID-19 patients with milder or more severe evolution. Moreover, a polygon connecting the outermost data points is shown for both group. As a single cluster of bacteria population was detected at both Phylum **(A)** and Family levels **(B)**, box plots were presented (instead of scatter plots).

Figure 2 graphically represents the distribution of *Z*-scores computed at clusters centroids at different taxa levels, showing that two clusters, composed by the linear combination of definite microorganisms residing within the gut of the patients at hospital admission, were able to significantly discriminate the clinical evolution of COVID-19 patients, in particular who will develop mild or severe respiratory involvement.

**Figure 3:**
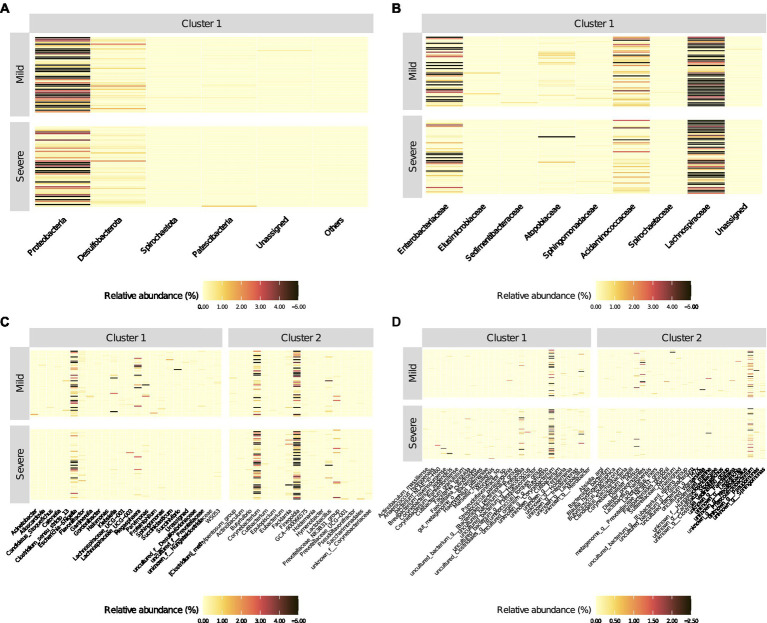
Heatmaps of relative abundance (%) of bacterial populations identified (into different clusters) by the PEnalized LOgistic Regression Analysis at different taxonomic hierarchy, grouped by COVID-19 patients with mild and severe symptoms, respectively. The order of the rows (patients) was determined by the main heatmap, which has been defined at the Phylum level. All other heatmaps are automatically adjusted according to the settings in the main heatmap.

At species levels, patients with mild symptoms were characterized by lower *Z*-scores from cluster 1 and higher *Z*-scores from cluster 2. Conversely, patients with “severe” outcome were categorized by higher *Z*-scores from cluster 1 and lower *Z*-scores from cluster 2. Moreover, Figure 3 reports the heatmaps shwing the relative abundance of each microorganism detected within each cluster at the phylum (Figure 3A), family (Figure 3B), genus (Figure 3C) and species (Figure 3D) level for each recruited subject in the good and severe group of outcome.

### Predicting the functional capabilities of microbial communities based on 16S datasets

**Figure 4:**
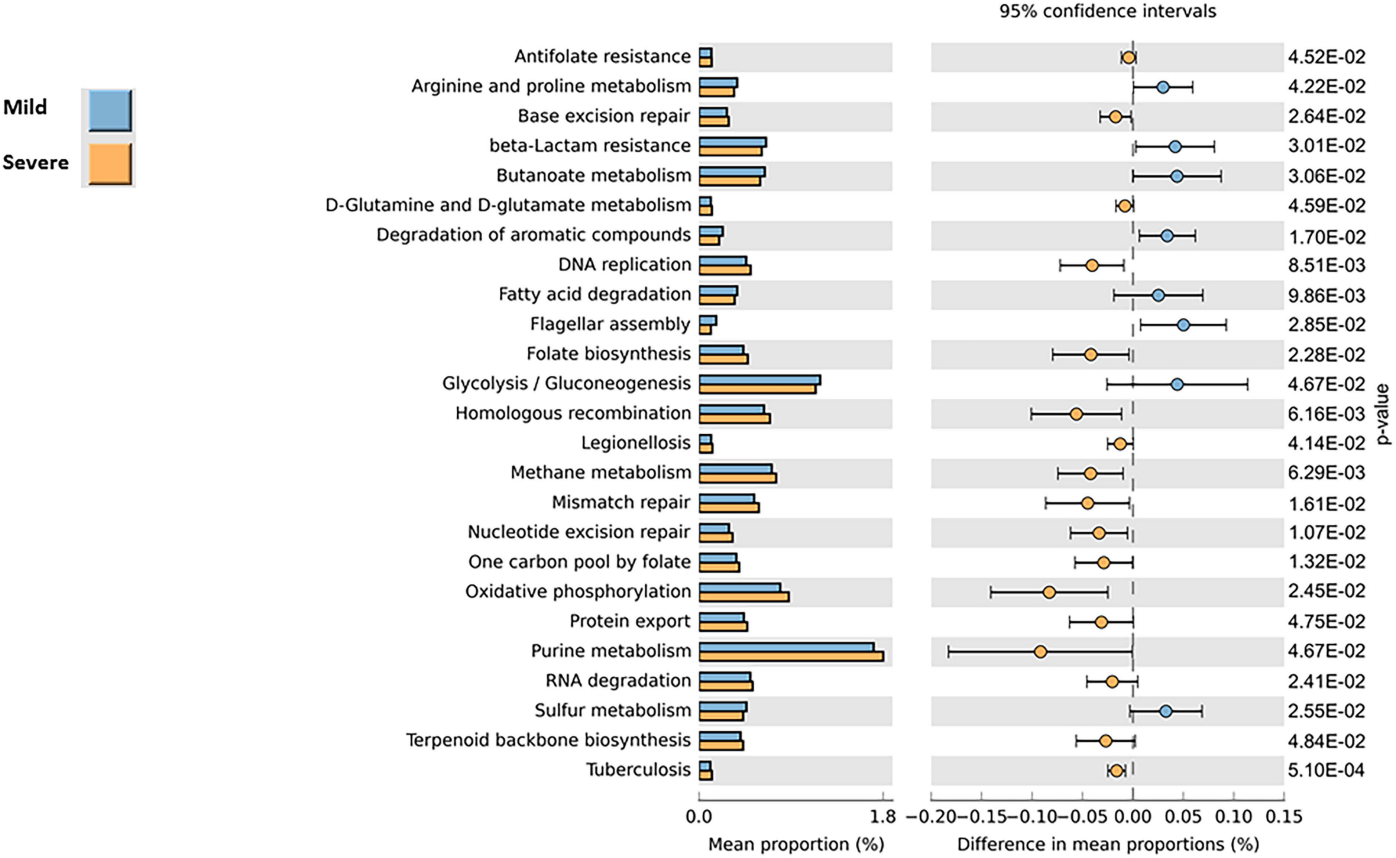
Prediction of the functional capabilities of microbial communities based on 16S datasets.

To explore the possible functional contributions of the gut microbiome on patients with mild or severe clinical evolution, we performed Tax4Fun2 prediction analyses based on 16S rRNA gene abundance profiles. As shown in Figure 4, functional profiling unveiled significant changes in the metabolic pathways between the two groups. At level 1 of analysis, we identified 25 the predicted differentially expressed metabolic pathways (*p*-values <0.05, Mann–Whitney test) that lead to the functional divergence in patients with mild or severe clinical evolution. Compared with the “severe” group, 8 functional categories were enriched in the gut microbiomes profiles of patients belonging to the “mild” group, including Arginine and Proline metabolism, beta_Lactam resistance, butanoate metabolism, degradation of aromatic compounds, fatty acid degradation, flagellar assembly, Glycolysis/Gluconogenesis, Sulfur metabolism. In contrast, in the gut microbiomes of severe group, 17 pathways were more abundant (i.e., Tubercolosis, Legionellosis, D-Glutamine and D-glutamate metabolism, DNA replication, folate biosinthesis, methane metabolism). Although these results show that in addition to microbial profile differences, there may be differences to functionalities of microbiome between mild or severe clinical evolution, it should be underlined that that functional prediction is only speculative and not necessarily indicative of actual observed differences.

## Discussion

Gut microbiota is a complex ecosystem which has been estimated to exceed 10^14^ microorganisms including bacteria, virus and fungi living in symbiosis with the host (Belkaid and Hand, 2014). This microbe community is useful in maintaining the host’s homeostasis since it influence several physiological functions, such as maintenance of the intestinal integrity, protection against pathogenic organisms, energy production and regulation of host’s immunity (Li et al., 2019). However, this status can become compromised following alterations in the core gut microbiota composition or functions, a condition known as dysbiosis (Hou et al., 2022). This change in intestinal microbiota profiles may compromise the host’s functions in which it is involved, including the immune system’s response against infections (Belkaid and Hand, 2014). In contrast, there is evidence that bacterial and viral infections can cause alterations in the gut microbiota profile, predisposing the host to secondary infections (not reported for the current cohort) or worsening clinical status (Harper et al., 2021; Kassa et al., 2021; Mizutani et al., 2022). In this context, an increasing importance is attributed to the role played by the intestinal microbiota in COVID-19 infection (Zuo et al., 2021). The bacterial dysbiosis in respiratory and intestinal tract is related to inflammatory response and although, mechanistically, this phenomenon remains poorly defined, the existence of the gut–lung axis and its implications in both health and disease may play a key role in both disease etiology and treatment. No significant differences were observed in the bacterial diversity of this study’s cohort, although previous studies have demonstrated that COVID-19 patients show a significantly reduced bacterial diversity (Mazzarelli et al., 2021; Hazan et al., 2022). This discrepancy may be explained because in the study by Hazan et al. (2022) and in our previous report (Mazzarelli et al., 2021) symptomatic patients were compared to healthy control, whereas in the current cohort, severely versus mild symptomatic hospitalized infected patients were compared.

Ferreira et al. (2020), reported a higher abundance of opportunistic bacteria such as *Streptococcus*, *Rothia*, *Veilonella*, and *Actinomyces*; and reduced levels of beneficial symbionts, including *Agathobacter*, *Fusicatenibacter*, *Roseburia*, and Ruminococcaceae. Accordingly, an increase in families containing potential pathogens such as Peptostreptococcaceae, Enterobacteriaceae, Staphylococcaceae, Vibrionaceae, Aerococcaceae, Dermabacteraceae, was observed in our previous paper when comparing the microbiota profiles of COVID-19 patients admitted to ordinary infectious ward to that of healthy controls (Mazzarelli et al., 2021).

Identifying prognostic markers is important in the management of COVID-19 patients to predict clinical evolution and it could also impact therapies (He et al., 2020). However, few studies evaluated the gut microbiota as a prognostic factor for disease progression in hospitalized patients. The existence of a correlation between the COVID-19 severity and fecal microbiota dysbiosis (including depletion of commensal bacteria) has been reported (De and Dutta, 2022). As shown by Zuo et al. (2020), twenty-three bacterial taxa were significantly correlated with disease severity, with the main bacteria belonging to the phylum Firmicutes and the genus *Coprobacillus*, as well as the *Clostridium ramosum* and *Clostridium hathewayi* species. In our cohort, the abundance of Firmicutes and of *Coprobacillus* genus was not significantly different between the two groups, whereas the species *Clostridium ramosum* and *Clostridum hatewayi*, which Zuo et al. found associated with COVID-19 severity, were not detected at all.

In the present study, analyzing gut microbiota composition in an association with other variables through multivariate analysis, we tried to identify a putative prognostic marker of clinical evolution in terms of severity of respiratory distress and need of ventilation support, useful to use early when the patient enters the hospital ward to predict the outcome. We grouped within the “severe” group patients requiring either non-invasive or invasive ventilation because they were all hospitalized in Intensive Care Unit, because other severe symptoms were diagnosed and considered and also because, although it would have been interesting to analyze the gut microbiota profiles separately in the two groups with invasive or non-invasive, the sample size would have been smaller, with the risk to mislead conclusions. We did not find significant differences in Chao1 and Shannon indices between the two groups of patients with different clinical evolution, and this evidence indicates that at baseline there is a uniformity of the microbiota in the patients admitted to the ordinary ward and is in agreement with our previous observation. However, fecal microbiota composition showed that, at phylum level, *Campilobacterota* and *Actinobacteriota* were increased in patients who had a more aggressive disease. Concerning *Campylobacterota*, they mainly include the genera *Campylobacter*, which are leading food-borne pathogens causing gastrointestinal infections, and Helicobacter, responsible of both gastric (such as gastritis, peptic ulcer, gastric cancer,) and extra-gastric (such as idiopathic thrombocytopenic purpura, anemias, MALT lymphoma, pancreatitis and pancreatic cancers) diseases (Nordestgaard et al., 2018). While for Helicobacter the standard clinical practice implies antibiotics-based therapies for bacterial eradication (Goderska et al., 2018), the *Campylobacter* infections, which in our study makes the greatest contribution to Campylobacterota increase, are often self-limiting and rarely require antibiotic treatment (Butzler, 2004).

Concerning *Actinobacteriota*, the family of Corynebacteriaceae was the main contributor to their increase in our study. Members of this family have been reported to cause alveolitis (Rintala, 2011), pneumonia (Yang et al., 2018), chronic respiratory diseases (Renom et al., 2014). Antibiotic therapy is recommended also in this case, although many species show multi-drug resistance, hence the need of selecting the appropriate antibiotic (Olender, 2013). Gradel et al. (2009), showed a possible association between the presence of *Campylobacter* spp. and inflammatory bowel disease and development of intestinal inflammation, so the finding of Campylobacter spp. in the gut microbiota of “severe” group of SARS-CoV-2 patients could explain the worst clinical outcome. Although finding genera containing pathogenic bacteria is not necessarily a good indicator of there being pathogenic species in the samples, all the six *Campylobacter* species identified in our samples have been described as involved in a number of inflammatory diseases. In detail: (i) *C. concisus* has been described as a pathogen associated with diseases of the gastrointestinal tract, such as Barrett’s esophagus, prolonged diarrhoea, and inflammatory bowel disease (Kirk et al., 2018); (ii) *C. gracilis* has been associated with periodontal diseases and pleuropulmonary infections (Shinha, 2015); (iii) *C. hominis* has been associated to ulcerative colitis and Crohn’s disease (Igwaran and Okoh, 2019); (iv) *C. showae* has been linked to gingivitis and periodontitis, has recently been associated with inflammatory bowel disease and colorectal cancer (Hsu et al., 2019); (v) *C. sputorum* has been described to cause diarrhoea in children (Lindblom et al., 1995); (vi) *C. ureolyticus* has been identified as a gastrointestinal pathogen, isolated from patients with Crohn’s disease and many other pathogenic conditions (Burgos-Portugal et al., 2012). The same is true for the *Corynebacterium* genus, which, with its 2.7 fold increase in the “severe” groups with respect to “mild,” gave the major contribution to Actinobacteriota increase. A number of *Corynebacterium* species identified in our cohort are known to be respiratory airways pathogens. In detail, just to cite few examples: (i) *C. argentoratense* has been detected in the upper respiratory tract of patients suffering from tonsillitis (Bomholt et al., 2013); (ii) *C. durum* is a pathogen of the respiratory tract (Riegel et al., 1997); (iii) *C.imitans* was first isolated in from a child suffering from acute respiratory disease that was initially diagnosed as pharyngeal diphtheria (Funke et al., 1997); (iv) *C. jeikeium* was reported to cause different forms of infections, including endocarditis, especially in immunocompromised subjects (Rezaei Bookani et al., 2017); (v) *C. propinquum* has been described in bronchopneumonia (Malkoçoğlu et al., 2016) and as the causative agent of respiratory tract infections worldwide (Jangda et al., 2016).

Furthermore, our results were in accordance to Farsi et al. (2022), who reported that alterations in gut microbiome were associated with COVID-19 severity and severe prognosis; such alterations included the increment of *Bacteroidota, Parabacteroides, Clostridium, Bifidobacterium, Ruminococcus, Campylobacter, Rothia, Corynebacterium, Megasphaera, Enterococcus, Aspergillus* spp. and the decrement of *Roseburia, Eubacterium, Lachnospira and Faecalibacterium.* In agreement with the findings by Farsi et al. (2022) *Campylobacter* and *Corynebacterium* were significantly increased while *Roseburia* and *Lachnospira* were under-represented in our COVID patients with severe disease compared to mild ones. On the contrary, Bacteroidota, *Parabacteroides*, *Bifidobacterium*, *Ruminococcus*, *Rothia*, *Enterococcus*, *Eubacterium*, *Faecalibacterium* were not differently represented between the two groups, while *Megasphaera* was decreased in the “severe” group. Finally, *Aspergillus* was not detected in our cohort. Several factor could explain such differences between our study and the review by Farsi et al. (2022), first of all the heterogeneity in the cohorts compositions, including different genetic backgrounds and lifestyles; in addition, methodological differences including DNA extraction matrix (stool sample rather than rectal swab), DNA isolation and sequencing procedure, data processing and statistical analysis.

In addition, a reduction at Family level of Ruminoccoccaceae was observed in patients with severe symptoms. A decreased abundance of Ruminococcaceae has been involved in several inflammatory bowel diseases and playing a main role in the maintenance of gut health due to their ability to produce butyrate and other Short Chain Fatty Acids (SCFAs) (Parada Venegas et al., 2019). Among SCFAs, butyrate owes antinflammatory properties that could explain why patients had a more severe disease. It should be noted that, unexpectedly, in our study, Enterobacteriaceae increased in the group of patients with a less severe outcome than in patients with a worse one. It could be argued that this phenomenon could be due to the higher exposure to active antibiotics (e.g., cephalosporins) more easily prescribed to most seriously ill patients at emergency department (76% of “severe” patients vs. 57% of “mild” patients were administered with antibiotics). Unfortunately data regarding antibiotic therapy before hospitalization were not avaible for all patients and it could have affected the different detection of these bacteria in the two groups of study patients. In the current study, Proteobacteria was more prevalent in less severe (mild) patients in the same line to what was observed by Yeoh et al. (2021) who described an increase in Proteobacteria (compared to COVID-19 negative control and COVID-19 patients during hospitalization) in the gut microbiota of COVID-19 patients discharged after negative RT-qPCR for viral RNA in nasopharyngeal swabs.

Furthermore, we took advantage of the Penalized Logistic Regression Analysis (PELORA) algorithm to identify the bacterial populations that best discriminate between the two groups and these changes in microbiota compositions differently affect the functional and metabolic profiles influenced by microbial communities belonging to patients with mild or severe clinical evolution.

Concerning the functional prediction of pathways affected by changes in gut microbial community, worth of note was among others, the increase in severely affected COVID-19 patients in Legionellosis and Tubercolosis, which both involves a pulmonary compromission, as COVID-19.

Being an observational, retrospective, single-centre study, our study has some limitations, including the enrollment of patients with different clinical management. This study require further testing and longitudinal data for them to be proposed as prognostic markers. Moreover, multiple factors can cause changes in microbiota, including the use of antibiotics. Noteworthy, that rectal swab was collected on the first day after patients’ hospitalization allowing us to limit this bias.

In conclusion, our study provides a list of bacterial markers more prevalent in severe patients compared to mild patients which may contribute to prognosis evaluation, paving the way to new interventional approaches.

## Data availability statement

Fastq raw sequencing data (deposited in ArrayExpress under the accession code E-MTAB-12236).

## Ethics statement

Ethical review and approval was not required for the study on human participants in accordance with the local legislation and institutional requirements. Written informed consent for participation was not required for this study in accordance with the national legislation and the institutional requirements.

## Author contributions

AM, MG, CP, LM, AVu, CG, and CF: sample and clinical collection. VP, CP, AVi, EB, GM, and NT: metagenomic experiments and analysis. AF, MC, and PP: statistical analysis. EN, FP, and VP: provision of study materials. VP, FP, EN, AM, and MG: writing – original draft. All authors: writing – review and editing. All authors contributed to the article and approved the submitted version.

## Funding

This study was supported by funds to the Istituto Nazionale per le Malattie Infettive (INMI) Lazzaro Spallanzani IRCCS, Rome, Italy, from the Ministero della Salute (Ricerca Corrente, linea 1; COVID-2020-12371817), the European Commission – Horizon 2020 (EU project 101003544 – CoNVat; EU project 101003551 – EXSCALATE4CoV; EU project 101005111 DECISION; EU project 101005075-KRONO) and the European Virus Archive – GLOBAL (grant nos. 653316 and 871029). SHARP: 848096. VP is supported by Italian Association for Cancer Research (AIRC) under IG 2019 – ID. 23006 project – P.I. and by Italian Ministry of Health Ricerca Corrente program 2022–2024.

## Conflict of interest

The authors declare that the research was conducted in the absence of any commercial or financial relationships that could be construed as a potential conflict of interest.

## Publisher’s note

All claims expressed in this article are solely those of the authors and do not necessarily represent those of their affiliated organizations, or those of the publisher, the editors and the reviewers. Any product that may be evaluated in this article, or claim that may be made by its manufacturer, is not guaranteed or endorsed by the publisher.

## Footnotes

^1^ https://www.illumina.com/content/dam/illumina-marketing/documents/products/other/16s-metagenomics-faq-1270-2014-003.pdf
