# Supplementary figures and images for: Gut microbiota composition in COVID-19 hospitalized patients with mild or severe symptoms
Source: Front Microbiol. 2022 Dec 6;13:1049215. doi: 10.3389/fmicb.2022.1049215 (PMC9763305; doi:10.3389/fmicb.2022.1049215)

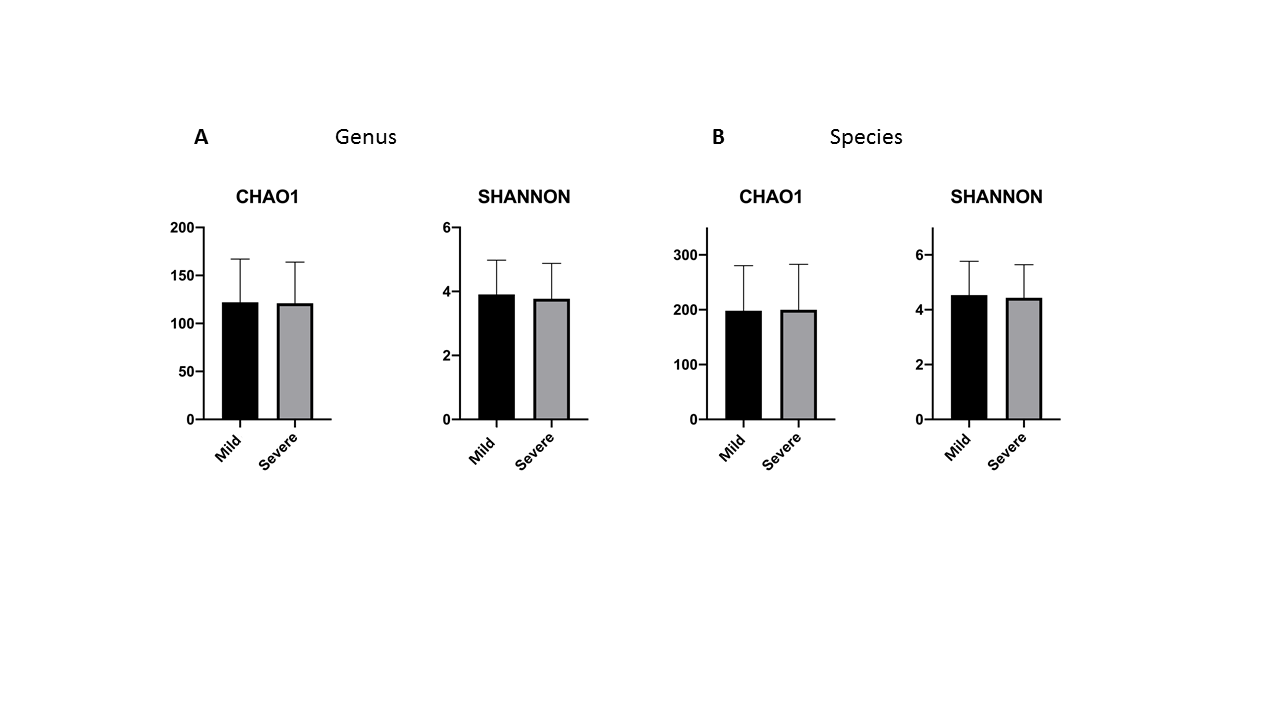

Supplement: Supplementary file 1 [file Image_1.TIF]

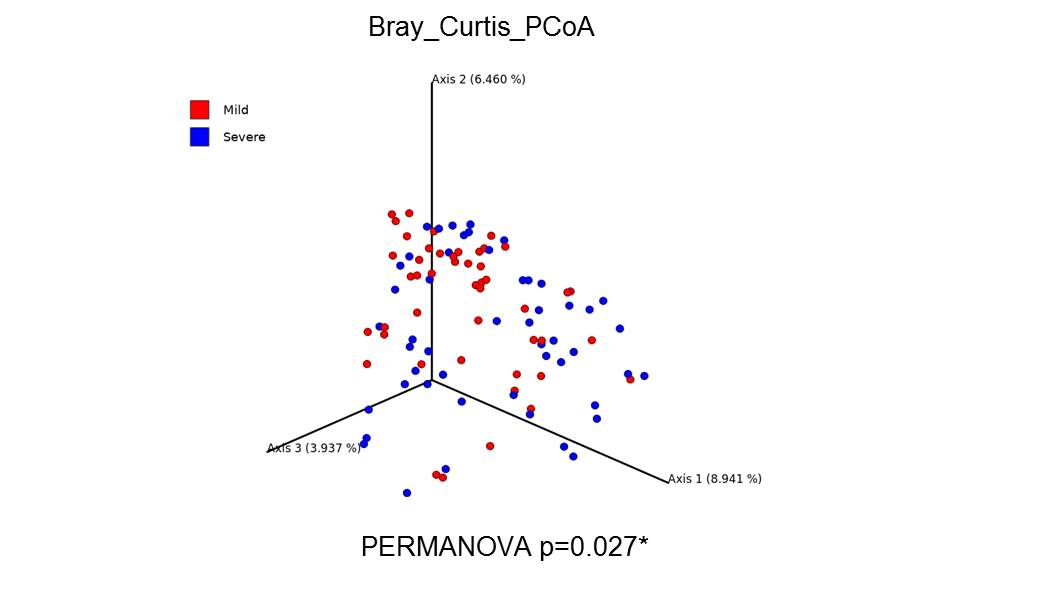

Supplement: Supplementary file 2 [file Image_2.JPEG]

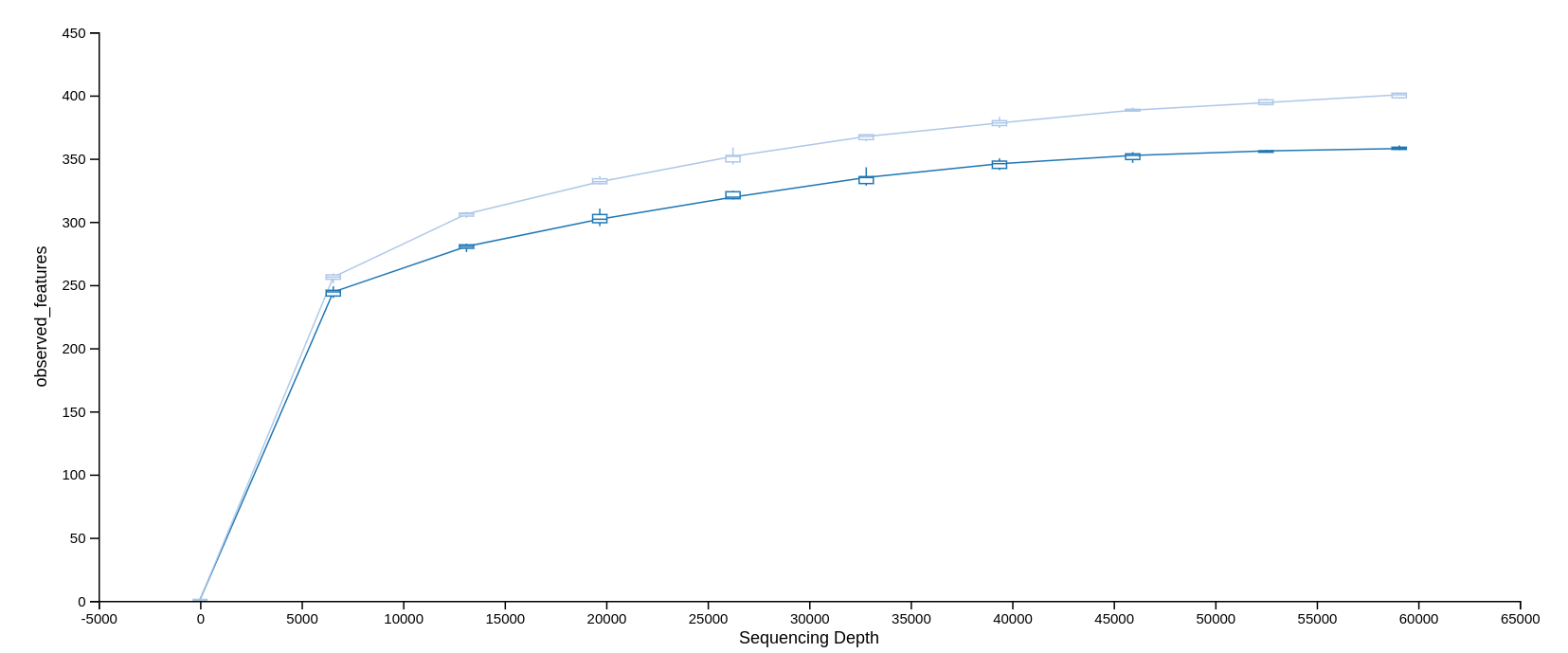

Supplement: Supplementary file 3 [file Image_3.PNG]
