# Supplementary material for: Gut microbiota composition in COVID-19 hospitalized patients with mild or severe symptoms
Source: Front Microbiol. 2022 Dec 6;13:1049215. doi: 10.3389/fmicb.2022.1049215 (PMC9763305; doi:10.3389/fmicb.2022.1049215)
Supplement: Supplementary file 5 [file Table_4.DOCX]

*Statistical analysis*

Demographic and clinical characteristics of COVID-19 patients with good (group “mild”) or severe (group “severe”) outcome were reported as mean ± standard deviation (SD), median along with interquartile range (i.e. first-third quartiles) and observed frequencies (and percentages) for continuous and categorical variables, respectively. For each continuous variable, the assumption of normality distribution was checked by means of quantile-quantile (Q-Q) plots and Shapiro-Wilk test. Comparisons between groups were performed by two-sample t-test and Chi-square test (or Fisher exact test as appropriate) for continuous and categorical variables, respectively. Stacked bar charts were used to show the fecal microbiota composition (i.e. mean relative abundance % [percentage/rate]) at phylum, family, genus, and species levels between COVID-19 patients with good (group “mild”) and severe respiratory impairment (group “severe”), respectively. The PEnalized LOgistic Regression Analysis (PELORA) was performed in order to identify clusters of bacterial populations, such that the linear combination of their abundances is differential between patients with good or severe outcome. This algorithm is mainly used to find predictive gene signatures from microarray data by using supervised grouping techniques. To this purpose, a standardized Z-score of each bacterium relative abundance (%) was computed as follows: as a first step, the abundance was logit transformed (i.e. computing the natural logarithm of the ratio between the relative abundance proportion and its complementary) and, as a second step, the logit transformed variable was standardized by subtracting its mean and dividing by its SD. When the relative abundance was exactly 0%, the logit transformation cannot be performed for that value and, to overcome this issue, such percentage was replaced by the minimum detectable one (i.e. 0.0003%) for the computation of Z-score only. Using PELORA algorithm, multiple clusters of bacterial populations can be detected. Each cluster has the characteristic that its centroid (i.e. the mean of the Z-scores of all identified bacteria within the cluster) was significantly higher (or lower) in one of the two compared groups (i.e. group “mild” and group “severe” patients). Two different free parameters must be set by the user in the PELORA algorithm: the number of centroids and the penalty parameter (λ). The number of centroids was set to varying between one and two, because we were mainly interested to detect no more than two informative pathways for each scenario whereas a number of different combinations of λ=(0,1/32,1/16,1/8,1/4,1/2,1) were evaluated, performing 200 bootstrap resampling of the data and recording the overall misclassification rate. For each specific scenario, the penalty parameter that achieved the lowest median misclassification rate (across the bootstrap samples) was chosen. Comparisons between Z-score means were assessed by two-sample t-test. Scatter plots (or box plots) of the Z-scores computed at cluster centroids as well as heatmaps of the relative bacteria abundance (%) identified by PELORA within each cluster were shown at phylum, family, genus and species levels. Two-sided p-values<0.05 were considered for statistical significance. All statistical analyses and plots were performed by the computing environment R (R Development Core Team 2008, version 4.2, packages: Complexheatmaps, supclust, ggplot2, gridExtra).
